# Effect of Medicine Adherence on the Occurrence of Cerebrovascular Disorders in Diabetes Mellitus Patients

**DOI:** 10.4178/epih/e2011001

**Published:** 2011-01-28

**Authors:** Il-Su Park, Hae-Sook Sohn

**Affiliations:** 1 Graduate School, Inje University, Busan, Korea.; 2 Department of Preventive Medicine, School of Medicine, Inje University, Busan, Korea.

**Keywords:** Diabetes mellitus, Cerebrovascular disorders, Medication adherence

## Abstract

**OBJECTIVES:**

To assess the association between the occurrence of cerebrovascular disorders and a medication adherence in diabetes mellitus patients.

**METHODS:**

Medical records from 1,114 new patients with diabetes mellitus were collected and the occurrence of cerebrovascular disorders was observed. Data was gathered from the health examination records of diabetes mellitus patients registered at the Korean Metabolic Syndrome Research from 1996 to 2005, medication records from the National Health Insurance Corporation and death data from the National Statistics Office from 1997 to 2007. Hazard ratios were analyzed using the Cox proportional hazard model to test the association between the occurrence of cerebrovascular disorders and the level of medication adherence. Medication adherence was calculated using Continuous measure of Medication Acquisition (CMA).

**RESULTS:**

Of 1,114 diabetes mellitus patients, cerebrovascular disorders occurred in 67 cases (6.1%). The mean duration for the development of a cerebrovascular disorder was 3.82 yr. Medication adherence (≥0.8 vs. <0.5: HR, 3.26; 95% CI, 1.47-7.21, ≥0.8 vs. 0.5-0.7 HR, 0.99; 95% CI, 0.33-2.95) was an independent factor associated with the occurrence of cerebrovascular disorders in diabetes mellitus.

**CONCLUSION:**

Increased medication adherence is necessary to prevent the occurrence of cerebrovascular disorders in diabetes mellitus patients. Furthermore we propose that CMA be considered as a method for monitoring medication adherence in clinics.

## INTRODUCTION

Most countries predict an increase in health care costs associated with the increasing occurrence of chronic diseases. In fact, rising health care costs are one of the most important social problems governments will face in the near future. Cancer, cardiovascular disease, cerebrovascular disorders, hypertension, and diabetes mellitus are among the most common chronic diseases around the world. In South Korea, a specific health care system for cancer has been established by the National Cancer Center Act, which led to the foundation of the National Cancer Center in 2000. In contrast, national health care policy regarding cardiovascular disease and cerebrovascular disorders, as well as hypertension and diabetes mellitus (both, major causal diseases for vascular diseases), have been addressed by the National project for cardiovascular and cerebrovascular disorders beginning in 2005. During its first term of activation (from 2005 to 2009), the construction of health care infrastructure was the main focus, and the strategies must be prepared for the 2nd term. Studies investigating practical methods for the management of risk factors of cardiovascular disease and cerebrovascular disorders would provide the evidence for the next stage of this National project.

Despite a decrease in death rate, cerebrovascular disorders continue to have the highest death rate in South Korea [1]. Therefore, research focusing on cerebrovascular disorders and related factors, especially modifiable risk factors, which can be targeted for intervention in health care programs, must be conducted. With the exception of age, a non-modifiable risk factor, modifiable risk factors for cerebrovascular disorders include: hypertension, diabetes mellitus, smoking cigarettes, alcohol consumption, and hyperlipidemia [2]. In South Korea, of these modifiable risk factors, the prevalence and death rate for diabetes mellitus have recently been on the increase [1, 3].

Moreover, because the medication adherence rate and blood sugar control rate remains low [3], population attributable risk of diabetes mellitus as the causal disease for cerebrovascular disorder occurrences is likely to increase. Hence, evaluation of the association between the occurrence of cerebrovascular disorders in diabetes mellitus patients and related risk factors is necessary. In addition to known risk factors for cerebrovascular disorders, medication adherence should be considered to be a modifiable risk factor in diabetes mellitus patients.

To date, in South Korea, no studies have been conducted on the association between the occurrence of cerebrovascular disorders in patients with diabetes mellitus and medication adherence.

The overall goal of this study is to understand the role of medication adherence in the prevention of cerebrovascular disorders in diabetes mellitus patients, by observing the association between the occurrence rate of this disorder in patients with diabetes mellitus and the level of patient medication adherence.

## MATERIALS AND METHODS

### Study Population

The study population were selected from 180,720 diabetes mellitus free individuals registered at the Korean Metabolic Syndrome Research Center from 1996 to 2005.

### Data Collection

I. Data for the occurrence of cerebrovascular disorders and medication adherence was collected from the National Health Insurance Corporation and the National Statistics Office from 1997 to 2007.

II. Risk factors for cerebrovascular disorders: Data for hypertension, cigarette smoking, alcohol consumption, total cholesterol, high-density lipoprotein (HDL), low-density lipoprotein (LDL), and triglyceride levels were collected from the records of health examinations conducted before the diagnosis of diabetes mellitus.

### Definition of Variables

#### Cerebrovascular disorder occurrences

Defined as those which resulted in death or a diagnosis of I60-I64 according to the ICD-10, and which occurred after the first diagnosis of diabetes mellitus.

#### Medication adherence

Medication adherence was observed using the Continuous measure of Medication Acquisition (CMA). CMA is considered to be the most appropriate measure of medication adherence for chronic diseases which require continuous medication therapy over an extended period of time, such as hypertension, diabetes mellitus, and mental diseases [4]. CMA is calculated as follows:

- The total number of days between the first and last prescriptions during the observation period is determined.
- From 'a,' the total number of days medication was dispensed is summed (except the last day, which is not included in 'a.').
- CMA is then calculated as 'b' divided by 'a' (b/a).

#### Age

Patient age at the time of diabetes mellitus diagnosis.

#### Cigarette smoking

Anyone who smokes, regardless of frequency or the duration for which he or she has been smoking.

#### Alcohol consumption

Anyone who consumes alcohol, regardless of the amount of alcohol consumed or the duration for which he or she has been drinking.

#### Hypertension

For the purposes of this study, hypertension and pre-hypertension (systolic BP ≥120 mmHg or diastolic BP ≥80 mmHg) are considered to be hypertension, according to the Seventh Report of the Joint National Committee on the Prevention, Detection, Evaluation, and Treatment of High Blood Pressure, JNC-7 [5].

#### Diabetes mellitus

Applying the standard of the American Diabetes Association (2007), a fasting blood sugar (FBS) of 126 or higher is defined as diabetic.

#### Diabetes mellitus occurrences

Cases representing major disease, in the range of E10-E14 from ICD-10 were defined as diabetes mellitus. For this study, criteria for diabetes mellitus occurrences were: a) Patients who visited a physician for diabetes mellitus for the first time between 1997 and 2007, after at least one prior health examination. b) Patients with no past history of diabetes mellitus, who had FBS levels <126 mg/dL, HbA1C levels ≤6%, and creatinine levels ≤1.5 mg/dL, based on their most recent medical examination prior to their first visit to a physician for diabetes mellitus. Of the patients who fulfilled criteria a) and b), patients whose first visit to the physician for diabetes mellitus was in the year 1997 were excluded from the study.

#### Duration of observation

The observation period began with the diagnosis of diabetes mellitus.

### Statistical Analysis

Hazard ratios (HRs) were analyzed using the Cox-proportional hazard model, with cerebrovascular disorder occurrences defined as 'event' and observation duration as 'time'. SPSS version 18.0 (SPSS Inc., Chicago, IL, USA) was used for statistical data analysis.

## RESULTS

The mean time for the development of cerebrovascular disorder in the study population was 3.82 yr, with no differences associated with gender, but a significant difference associated with age (3.98 yr for patients ≤54 yr of age, and 3.64 yr for patients ≥55 yr of age) (Table 1).

Of the 1,114 diabetes mellitus patients included in the present study, 67 (6.0%) were later diagnosed with cerebrovascular disorders. HRs for the occurrence of cerebrovascular disorders in diabetes mellitus patients were calculated to be 1.90 (95% CI, 1.18-3.07) for male vs. female patients, and 2.02 (95% CI, 1.22-3.33) for patients ≤54 yr old vs. patients ≥55 yr old (Table 2).

HRs for all risk factors identified before the occurrence of diabetes mellitus (cigarette smoking, alcohol consumption, hypertension, total cholesterol, HDL, LDL, and triglycerides), were not statistically significant (Table 3).

Medication adherence (≥0.8 vs. <0.5: HR, 3.31; 95% CI, 1.50-7.33) and patient age (patients ≤54 yr old vs. patients ≥55 yr old: HR, 1.81; 95% CI, 1.08-3.03) were both found to be statistically significant independent factors associated with cerebrovascular disorder (Table 4).

**Figure 1:**
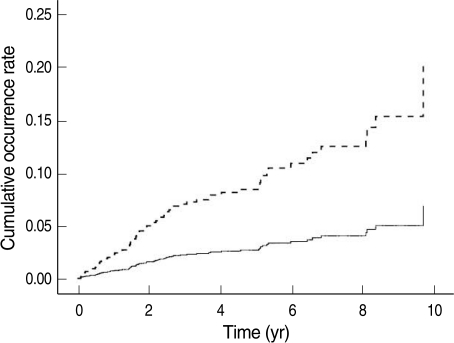
Cumulative occurrence rate of cerebrovascular disorder versus level of medication adherence in diabetes mellitus patients. Medicaton adherence; —, ≥8; –·–, 5-7; ----, <5.

Figure 1 shows the cumulative rate of cerebrovascular disorder occurrences versus medication adherence. On the graph, traces corresponding to medication adherence ≥0.8, and between 0.5-0.7 overlap and are difficult to differentiate.

## DISCUSSION

The increased occurrence of chronic diseases, which is affected by the population growth of people 65 yr of age and older, lifestyle changes and environmental changes, is an important issue that modern society must take seriously. In addition, the population growth in South Korea of people aged 65 and older is faster than in any other country, accelerating the rate at which our society is burdened by such phenomenon.

This burden can be significantly lessened by preparing specific plans for the prevention, treatment and rehabilitation of major chronic diseases. Although the mortality rate from cerebrovascular disorder is on the decrease, it still constitutes 10% of the total causes of death, and has the highest mortality rate among single diseases. Deaths due to diabetes mellitus (a risk factor for cerebrovascular disorder) are also on the increase, and currently this disease has the 6th highest mortality rate [1].

With respect to cerebrovascular disorder occurrences in diabetes mellitus patients, for every 1% increase in HbA1C levels, there is a 1.37 fold increase in the mortality rate [6], whereas for every 1% decrease in HbA1C, there is a 12% decrease in the mortality rate due to cerebral infarction [7].

In contrast, in patients with no past history of cerebrovascular disorders, it has been reported that blood sugar level control has no effect on the occurrence of cerebrovascular disorders [8], indicating a need for further study of the association between diabetes mellitus care and the occurrence of cerebrovascular disorders.

In light of this, the present study investigated the association between medication adherence of diabetes mellitus patients and cerebrovascular disorder occurrences.

The mean time for the development of cerebrovascular disorders in diabetes mellitus patients was 3.82 yr (SD 2.53 yr), with no differences associated with gender. In addition, although the occurrence rate was not different with respect to gender, the rate was substantially lower for patients ≤55 yr old compared to patients ≥55 yr old. In this study, absence of a past history of diabetes mellitus was confirmed using health examination records from before the initial diagnosis of diabetes mellitus, and medication data from 1997 to 2007, which excludes any past history of diabetes mellitus before 1997. Thus, the mean time before the development of cerebrovascular disorders in diabetes mellitus patients could be underestimated.

According to observations based on data from health examinations conducted prior to initial diagnosis of diabetes mellitus, all other risk factors (cigarette smoking, alcohol consumption, hypertension, total cholesterol, HDL, LDL, and triglyceride levels) were found not to be associated with the occurrence of cerebrovascular disorders.

In addition to being a known as a risk factor for cerebrovascular disorders, diabetes mellitus is also associated with an increased recurrence risk [9, 10]. Moreover, some studies have shown that the association between diabetes mellitus and cerebrovascular disorders differs depending on the type of cerebrovascular disorder. For example, although diabetes mellitus is a risk factor for the occurrence of cerebral infarction [11-13], whether it is a risk factor for cerebral hemorrhage is, reportedly, unclear [12, 14]. In fact, in the present study, of the 67 patients diagnosed with a cerebrovascular disorder, 12 patients had cerebral hemorrhages, while 50 suffered from a cerebral infraction. However, because these two diseases are not currently differentiated in the National health care system, the present study analyzed overall occurrences and causes of cerebrovascular disorders. We propose further study on the association between diabetes mellitus and different types of cerebrovascular disorders.

Although the occurrence of cerebrovascular disorders in the general population is higher in the elderly [2], in diabetes mellitus patients younger than 55, the risk of cerebrovascular disorder is 10 times [15]. However, in the present study, patients 55 and older were 1.81 times more likely to develop cerebrovascular disorders than patients less than 55 yr old, suggesting that additional study on the association between age and the occurrence of cerebrovascular disorder in diabetes mellitus patients is necessary.

The association between blood sugar level control in diabetes mellitus patients and a reduced risk for cerebrovascular disorders has yet to be clearly determined [2, 16].

In this study, medication adherence was used as an index for observing the association between diabetes mellitus care and the occurrence of cerebrovascular disorders. Although the observation of the total days of prescription from medical insurance data were only used in this study to measure patients' compliance with their prescription, this data is also able to quantitate medication adherence in clinics and therefore, medication adherence can be used as an index to monitor diabetes mellitus care.

Measurement of patient compliance (medication adherence) using refilled prescription data from the drugstore is most appropriate for large scale studies using secondary data [4]. The present study used the Continuous measure of medication acquisition (CMA), the most appropriate measure of medication adherence in chronic diseases that require long-term medication. CMA is also used in medication adherence observations by the National Health Insurance Corporation, and a CMA of 0.8 is used as a reference for observing patient compliance [17]. Because there is no nationally suggested level of medication adherence, CMA levels of 0.8 and 0.5, in three divisions were used in the present study.

Hazard ratios for cerebrovascular disorder occurrences adjusted for gender and age were statistically significant for medication adherence ≥0.8 vs. <0.5 (HR, 3.31; 95% CI, 1.50-7.33), but were not significant for medication adherence ≥0.8 vs. 0.5-0.7 (HR, 0.99; 95% CI, 0.34-2.99). Therefore, ensuring a medication adherence of at least 50% or more may reduce the risk of cerebrovascular disorder in diabetes mellitus patients. In the present study, there are several limitations, such as the possible underestimated time elapsed before patient development of cerebrovascular disorders, and the accuracy of diagnoses from medical insurance data, suggesting that the level of medication adherence required for preventing cerebrovascular disorders in diabetes mellitus patients is not definite.

However, observation and control of medication adherence based on CMA monitoring is recommended, and further study on the level of medication adherence required for the prevention of cerebrovascular disorders in diabetes mellitus patients is suggested.

## Figures and Tables

**Table 1:**
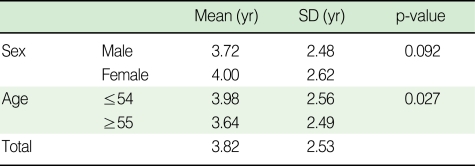
The mean duration from initial diagnosis of diabetes mellitus to the occurrence of a cerebrovascular disorder by sex and age

SD, standard deviation.

**Table 2:**
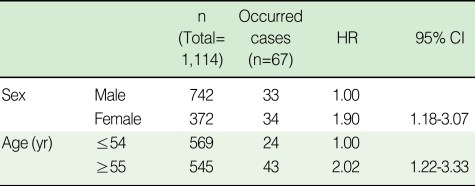
Crude hazard ratios (HRs) for the occurrence of cerebrovascular disorders in diabetes mellitus patients versus sex and age

**Table 3:**
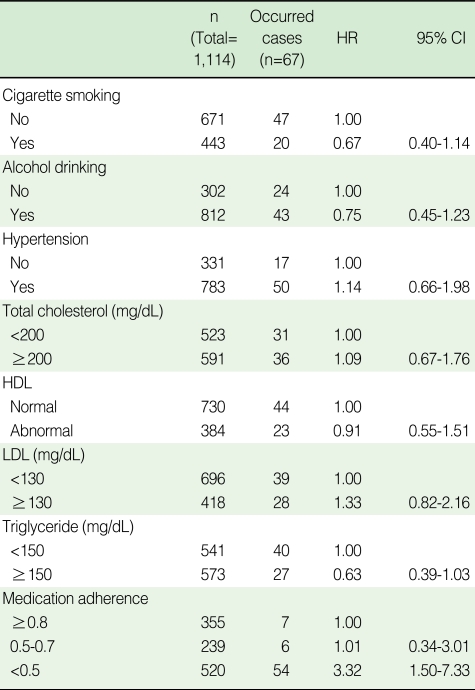
Crude hazard ratios (HRs) for the occurrence of cerebrovascular disorders in diabetes mellitus patients versus modifiable risk factors

HDL, High density lipoprotein (mg/dL); male-normal ≥40, abnormal <40, female-normal ≥50, abnormal <50; LDL, Low density lipoprotein.

**Table 4:**
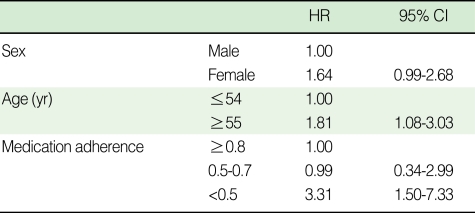
Adjusted hazard ratios (HRs) for the occurrence of cerebrovascular disorders in diabetes mellitus patients^*^

^*^Cox-proportional hazard model included gender, age, and medication adherence as covariates.
